# pHoenix score: development and validation of a novel approach to decrease the number of inconclusive GERD diagnoses

**DOI:** 10.1007/s00464-024-11105-1

**Published:** 2024-08-27

**Authors:** Andrés R. Latorre-Rodríguez, Sumeet K. Mittal, Hailey Simmonds, Peter Kim, Ross M. Bremner

**Affiliations:** 1 Norton Thoracic Institute, St. Joseph’s Hospital and Medical Center, 500 W Thomas Road, Phoenix, AZ 85013 USA; 2 Grupo de Investigación Clínica, Escuela de Medicina y Ciencias de la Salud, Universidad del Rosario. Bogotá D.C., Bogotá, Colombia; 3 Creighton University School of Medicine, Phoenix Health Sciences Campus, Phoenix, AZ USA; 4 School of Molecular Sciences, Arizona State University, Tempe, AZ USA

**Keywords:** Gastroesophageal reflux, GERD, Reflux, Ambulatory esophageal pH monitoring, PH monitoring

## Abstract

**Background:**

The Johnson–DeMeester composite score (DMS) is the historical gold standard for diagnosing gastroesophageal reflux disease (GERD). The Lyon Consensus outlines criteria for diagnosing GERD by pH monitoring, defining normal acid exposure time (AET) as < 4% and pathological as > 6%, presenting diagnostic uncertainty from 4 to 6%. We aimed to (i) calculate the proportion of borderline studies defined by total AET alone that are reclassified as normal or pathological by the DMS, (ii) determine the importance of supine AET for reclassification, and (iii) propose a new classification system using a composite score that considers positional changes.

**Methods:**

This single-center, retrospective, observational study analyzed data from patients with an overall total AET from 2 to 6% on 48-h pH monitoring (Bravo pH capsule). Preselected predictors (supine and upright AET) were included in a model to create a composite score (i.e., pHoenix score) using the regression coefficients. The model was internally validated, and discriminative ability was tested against the DMS and compared to the total AET.

**Results:**

We identified 114 patients (80 [70.2%] women; median age, 55 years). Using the total AET, 26 (22.8%) were classified as normal and 88 (77.2%) as borderline; however, using the DMS, 45 (39.5%) were classified as normal and 69 (60.5%) as pathological. The new pHoenix score demonstrated strong discriminative ability (AUC: 0.957 [95% CI 0.917, 0.998]) with high sensitivity and specificity (lower threshold, 94.4% and 79.2%; upper threshold, 87 and 95.8%). Compared to the total AET alone, the pHoenix score significantly decreased the proportion of inconclusive cases (77.2% vs. 13.2%, *p* < 0.001).

**Conclusion:**

Total AET has low sensitivity to identify pathological reflux as it disregards supine versus upright reflux. The pHoenix score improves the distinction between normal and pathological cases and reduces ambiguity, offering an alternative approach to diagnosing GERD that addresses the limitations of using total AET alone or the DMS.

**Graphical abstract:**

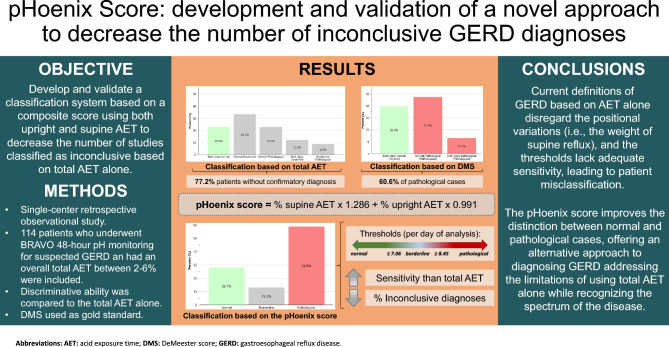

**Supplementary Information:**

The online version contains supplementary material available at 10.1007/s00464-024-11105-1.

Gastroesophageal reflux disease (GERD) is a multifactorial chronic condition affecting ~ 20% of the adult population of the United States [1]. The underlying pathophysiology involves an incompetent lower esophageal sphincter complex that normally prevents the retrograde flow of gastric contents across the pressure gradient into the distal esophagus [2–4], leading to esophageal or extraesophageal symptoms and complications [5, 6].

Traditionally, the diagnosis of GERD has been based on classic symptomatology (i.e., presence of heartburn or regurgitation) and response to medical therapy; however, this method is inaccurate and cannot be used as a guide for determining treatment (i.e., surgical or endoscopic procedures). In 1974, a seminal paper by Johnson and DeMeester [7] introduced the Johnson–DeMeester score (DMS), which established normal and abnormal values for distal esophagus acid exposure based on ambulatory transnasal, catheter-based, 24-h pH monitoring; thus heralding a new era of scientific interest in the objective diagnosis of GERD. Despite limitations of the testing procedure, including the need for manometry for catheter placement, patient discomfort, and limitation of daily activities, the DMS remains the gold standard for the definitive diagnosis of GERD [7–9].

The introduction of the Bravo pH capsule (Medtronic, Minneapolis, MN), a wireless endoscopically placed system for pH monitoring, circumvents multiple issues with conventional transnasal pH monitoring [10, 11]. The Bravo capsule has increased patient and physician acceptance of objective testing, and the distal esophageal acid exposure can be assessed beyond 24 h, potentially increasing the accuracy of the test [10, 12–14]. However, using the total acid exposure time (AET) thresholds of < 4 and > 6% does not distinguish between upright and supine reflux, which is an important component of the DMS. In addition, multiday testing introduced a new conundrum as the AET can be normal or abnormal on different days during the test. The recently introduced Lyon Consensus v2.0 [13] further added to the confusion by creating a “borderline” definition of distal esophageal AET that ultimately increases the number of patients with an inconclusive GERD diagnosis.

We hypothesize that a new score giving increased weight to supine exposure (i.e., preserving the core of the DMS) but incorporating the advantages of prolonged, wireless testing will address the diagnostic dilemma within the gray area (i.e., borderline total AET) [8] to help establish optimal management (i.e., surgical or medical) and address the current gap in providing a definitive diagnosis (possibly reducing the overuse of proton pump inhibitors [PPIs] in patients without GERD) [14]. In this study, we aimed to (i) measure the proportion of borderline studies defined by total AET alone that are reclassified as normal or pathological based on the DMS, (ii) determine the importance of supine AET for reclassification and (iii) develop and validate a classification system based on a composite score (i.e., pHoenix score) using both upright and supine AET to decrease the number of studies classified as inconclusive.

## Methods

### Study design and source of data

**Fig. 1:**
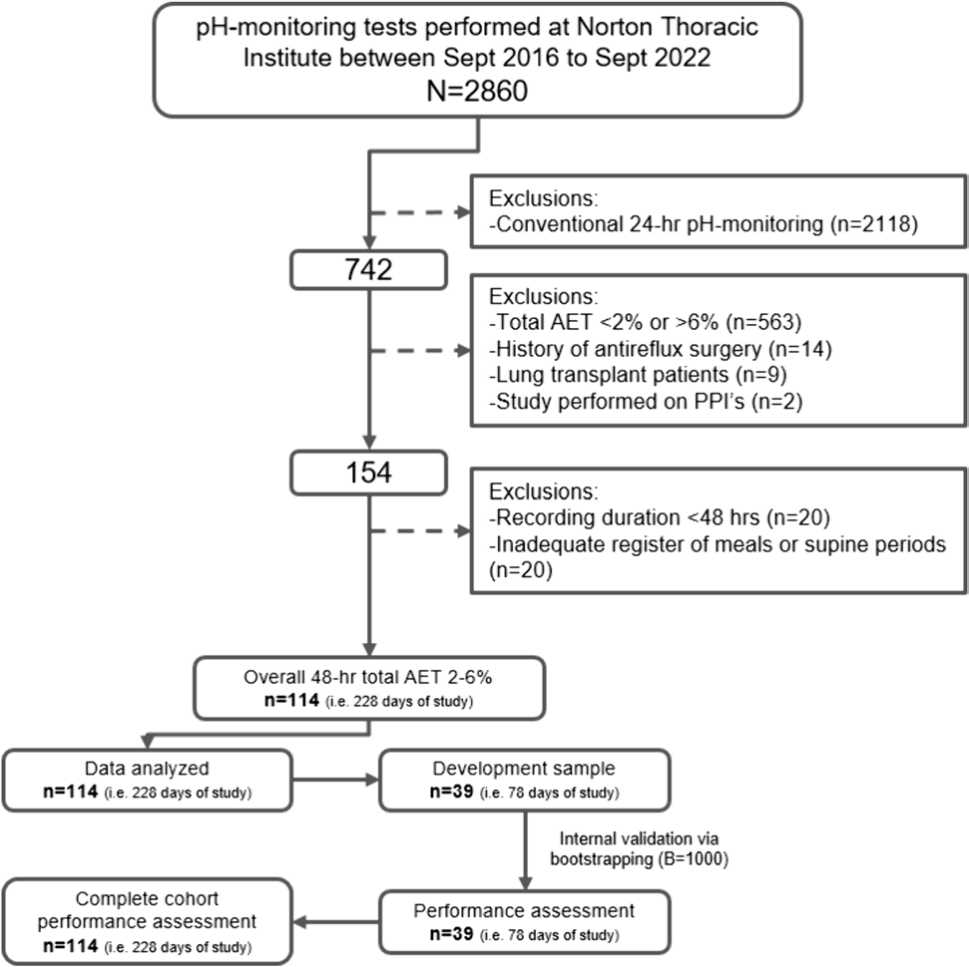
Study flow chart. *PPI* proton pump inhibitor

This single-center, retrospective observational cohort study analyzed de-identified data collected from a prospectively maintained database of all patients who underwent esophageal functional testing (i.e., high-resolution manometry [HRM] or pH monitoring) between September 2016 and September 2022 at the Norton Thoracic Institute (NTI), Phoenix, AZ. The database contains basic demographic and clinical data as well as the most commonly used physiological and diagnostic parameters of the functional assessments; additional data including relevant endoscopic findings were extracted from the patient’s charts. The NTI research committee and the Institutional Review Board of St. Joseph’s Hospital and Medical Center, Phoenix, AZ, approved this study under the Foregut Umbrella Protocol (PHXU-21-500-136-73-18, date: 03-Apr-2023). Written patient consent was waived due to the nature of the study design. The Transparent Reporting of a multivariable prediction model for Individual Prognosis or Diagnosis (TRIPOD) statement and checklist were followed to ensure transparency of the results and overall quality of the manuscript (Supplementary Material S1). The study flow diagram is presented in Fig. 1.

### Study population

The cohort included patients who underwent 48-h pH monitoring for evaluation of suspected GERD using the wireless Bravo capsule with an overall 48-h total AET between 2 and 6% regardless of endoscopic findings or HRM results (if done). Patients with a history of antireflux surgery, lung transplantation, achalasia, or esophagogastric outflow obstruction and those who did not adequately register meals and supine periods were excluded; studies that were not performed off acid suppression therapy long enough (i.e., PPIs < 7 days or H2-antagonist < 3 days) were also excluded.

### Procedures

#### pH monitoring and endoscopic assessment

The Bravo capsule system was inserted transorally with endoscopic guidance and positioned 6 cm above the endoscopic gastroesophageal junction. All Bravo capsule placements and esophagogastroduodenoscopy (EGD) procedures were performed under sedation using a standard video endoscope (Olympus, Shinjuku City, Tokyo, Japan) by a foregut surgeon. At our center, all patients are routinely instructed to register each meal and supine period using both the electronic device as well as a written diary. We generally perform 48-h pH monitoring; however, if longer periods were monitored, then only the first 48 h (i.e., day 1 and 2) were analyzed for this study. Evidence of hiatal hernia, Barrett’s esophagus, peptic strictures, and the grade of erosive esophagitis were retrieved from EGD reports.

#### High-resolution manometry

Esophageal manometry (if done) was performed in most cases within 7 days of pH monitoring. A 36-channel catheter with circumferential solid-state pressure transducers was placed at 1-cm intervals (Medtronic, Minneapolis, MN). The esophageal body peristalsis, as well as the function of the esophagogastric junction, were assessed following the Chicago Classification (CC) system [15].

### Definitions

#### Pathological reflux based on Johnson–DeMeester score (DMS)

Calculation of the DMS and the diagnostic thresholds have been described elsewhere [7, 16]. When evaluating each 24 h of the registry individually (i.e., a study day), a DMS ≥ 14.73 was considered pathological. For this study, when evaluating 48 h of registry, a DMS score ≥ 14.73 on either day of the study was considered pathological, and both days with a DMS < 14.73 was considered normal, as suggested by Ayazi et al. [12].

#### Pathological reflux based on total acid exposure time (AET)

Definitions and thresholds were adapted from the Lyon Consensus 2.0 [13]. When evaluating 24 h of registry (i.e., a study day), a total AET > 6% was classified as conclusive pathological reflux, between 4 and 6% as borderline/inconclusive, and < 4% as normal acid exposure. For this study, when evaluating 48 h of registry, both days with an AET > 6% was classified as pathological reflux; both days with a total AET < 4% was classified as normal; and the remaining scenarios (i.e., both days with borderline AET or one day with borderline AET and the other with a total AET of < 4% or > 6%) were classified as inconclusive.

### Outcomes and predictors

For model development, the DMS was used as the dependent variable, and the performance of the proposed score was assessed against the DMS definitions (i.e., historical gold standard) and compared in parallel with the total AET. Potential predictors (i.e., supine AET and upright AET) of the outcome were preselected based on clinical considerations and published studies reporting that patients presenting a predominant supine acid exposure have more frequent mucosal injury and a higher likelihood of more severe GERD-related esophageal complications than patients with predominant upright acid exposure [17, 18].

### Sampling and sample size

All consecutive individuals who underwent wireless pH monitoring at our center and had data available in the study database were included in the cohort if the inclusion criteria were met. Therefore, a maximum available sample size was obtained by convenience.

### Missing data and sensitivity analysis

The queried database included the overall pH test metrics for 48 h of registry (e.g., average DMS, total AET, supine AET, upright AET) but not the individual parameters for each study day; thus, all studies were reanalyzed by two authors (A.L. and H.S.) to obtain the missing values. Furthermore, the endoscopic findings were not recorded during routine data input into the esophageal functional testing database, thus, chart extraction was required. Importantly, a sensitivity analysis was not required to compare the performance and discrimination ability of the proposed score because the included predictors were complete for all individuals; hence, a complete case analysis was performed.

### Data analysis

Data were reviewed for completeness and inconsistencies; if inconsistent or missing values were identified, they were corrected or completed as described above. Categorical variables are expressed as count and proportion, and continuous data are expressed as median and interquartile range (IQR) or mean and standard deviation (SD). All continuous variables were assessed for normal distribution using the Shapiro–Wilk test if *n* ≤ 50 or the Kolmogorov–Smirnov test if *n* > 50. Nonparametric correlations were assessed using Spearman’s rank coefficients and plotted as appropriate. To assess if a misclassification occurred when using the total AET alone compared to the DMS among the cohort, cross-tabulations were performed, and differences between the diagnostic categories were assessed using the Chi-square test.

To evaluate if the preselected predictors (i.e., upright AET and supine AET) caused a difference in diagnostic categories between the DMS and the total AET diagnostic definitions, we conducted a descriptive analysis of individual days of pH monitoring across the cohort. Differences in supine and upright AET were assessed using the Mann–Whitney *U* test or the Kruskal–Wallis test as appropriate. Further, we identified patients with “incongruent” study days (i.e., days of analysis with a normal or borderline total AET but pathological DMS), and this subgroup of patients formed the development sample.

Thereafter, we employed the data from individual days of analysis from the development sample to propose a new scoring system using the preselected predictors. To better understand the role and confirm the relative weight of supine AET as an explanatory parameter of the DMS, a marginal model was created with this variable only; then, an additive linear regression model was created, including both preselected predictors, and the resulting equation using the regression coefficients was established as the new score calculation method. Importantly, the intercept was preserved in the model development but excluded from the equation knowing a priori that it should be zero (i.e., considering that at a population level, the spectrum of results ranges from utterly normal to very pathological rather than presenting only borderline results, also because a DMS near to 0 correlates with acid exposure time close to 0%). The collinearity was assessed using the minimum tolerance and the variance inflation factor (VIF). The model’s prediction was internally validated using simple bootstrapping with samples of the same size as the original development sample (*B* = 1000), and the overall performance of the model was assessed using the adjusted R^2^ and the quality with the Akaike information criterion (AIC).

The ability of the new score to discriminate between pathological acid exposure and physiological exposure was quantified as the area under the receiver operating characteristic curve (AU-ROC). The AU-ROC and the maximum difference between cumulative distribution functions (i.e., via Kolmogorov–Smirnov test) were tested against the DMS and compared with the total AET. Using Youden’s index, the optimal lower and upper thresholds (i.e., cutoffs) of the new score were selected, aiming to have the best ability to discriminate between true negative and true positive cases. Thereafter, the performance of the score was compared again to the standard reference and validated within the entire cohort using the same methods. The sensitivity (SE), specificity (SP), positive predictive value (PPV), and negative predictive value (NPV) were calculated for each threshold. Finally, a diagnostic classification system using both days of registry based on the new score was proposed and compared to the categories using the total AET. The alpha (*α*) level was set at 0.05, and SPSS v29.0 (IBM SPSS Statistics; Armonk, NY) was used for all statistical analyses.

## Results

### Baseline characteristics and pH metrics of the entire cohort and the development cohort

A total of 114 patients who underwent 48-h wireless pH monitoring with an overall total AET between 2 and 6% were included in this cohort; thus, 228 study days were retrieved for statistical analyses. Most of the subjects in this cohort were female (*n* = 80 [70.2%]); the median age and body mass index were 55 years and 28.9 kg/m^2^, respectively. A total of 57 patients had an overall normal total AET (i.e., < 4% AET over 48 h), whereas 57 had an overall borderline total AET (i.e., 4–6% AET over 48 h). Table 1 summarizes the baseline characteristics of the entire cohort and the development cohort as well as the main pH parameters by day of study.

**Table 1:** Baseline demographic and clinical characteristics of the entire cohort and the development sample

	Entire cohort (*n* = 114)	Development sample (*n* = 39)
Demographics		
Age, years, median (IQR)	55 (44–66)	51 (41–64)
Sex, female, no (%)	80 (70.2)	24 (61.5)
BMI, kg/m^2^, median (IQR)	28.9 (24.8–32.4)	29.5 (25–32.8)
Endoscopic and manometric characteristics, no (%)		
Endoscopic evidence of GERD	10 (8.8)	6 (15.4)
Erosive esophagitis LA ≥ B	3 (2.6)	2 (5.1)
Suspected Barrett’s esophagus	7 (6.1)	4 (10.3)
Esophageal peptic stricture	0 (0)	0 (0)
Evidence of hiatal hernia	44 (38.6)	14 (35.9)
Hiatal hernia size > 2 cm	29 (25.4)	8 (29.6)
Manometric diagnosis of IEM or AC*	20 (30.8)	8 (29.6)
pH-monitoring metrics, mean (SD)		
Day 1		
DeMeester score	12.41 ± 7.2	17.01 ± 5.1
Total AET (%)	3.5 ± 1.9	4.5 ± 1.3
AET during upright position (%)	4.5 ± 3	5.4 ± 3.5
AET during supine position (%)	2 ± 3	3.7 ± 3.6
Number of reflux episodes	22.3 ± 13.5	26 ± 13.1
Duration of longest reflux episode, min	14 ± 12.8	19.6 ± 12
Day 2		
DeMeester score	14.3 ± 8.4	16.0 ± 6.7
Total AET (%)	4.2 ± 2.3	4.4 ± 1.2
AET during upright position (%)	5.9 ± 42	6.3 ± 3.5
AET during supine position (%)	2.2 ± 4.2	3 ± 5.7
Number of reflux episodes	26.5 ± 18.7	27.4 ± 16.8
Duration of longest reflux episode, min	12.9 ± 11.5	12.9 ± 9.6

*AC* absent contractility, *BMI* body mass index, *IEM* ineffective esophageal motility, *LA* Los Angeles grade of erosive esophagitis, *SD* standard deviation

*Manometric data was available for 65 patients for the entire cohort and 27 for the development sample

### Misclassification of borderline studies using total AET alone

**Fig. 2:**
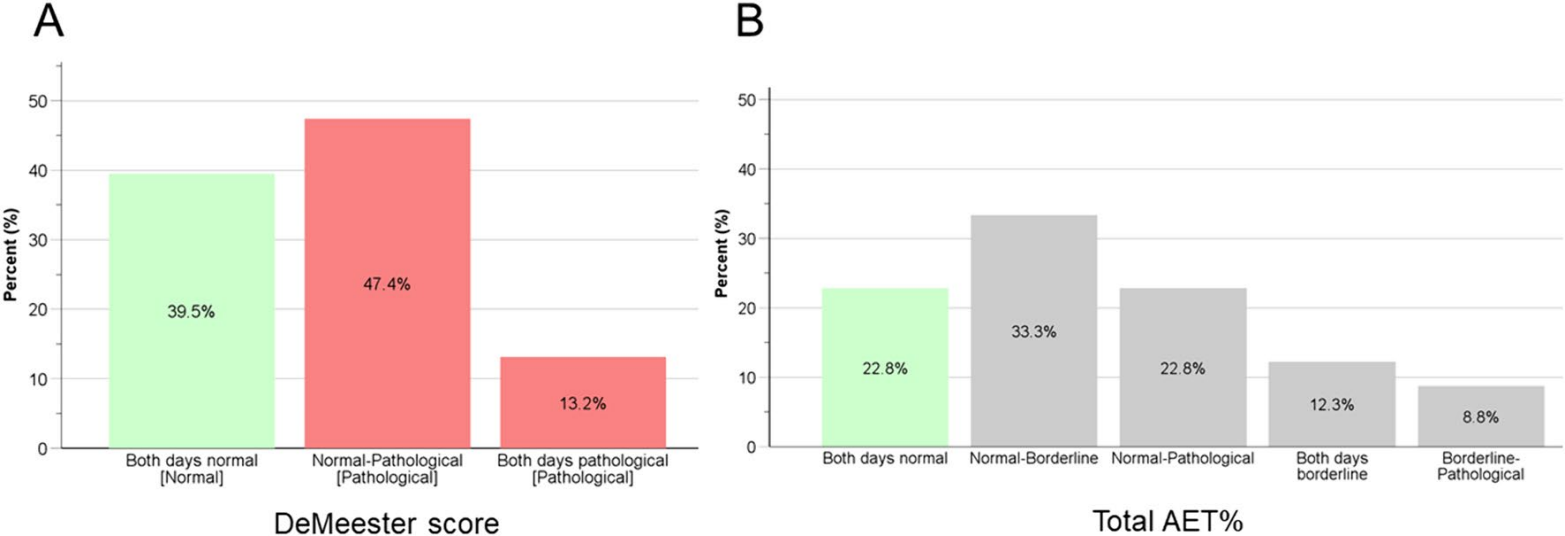
Proportion of patients falling into each diagnostic category according to **A** DeMeester score and **B** percentage of total acid exposure time (AET)

Among the cohort, 26 (22.8%) patients had a normal total AET (i.e., < 4%) based on the Lyon Consensus criteria [13] on both days; however, the remaining 88 (77.2%) had at least one pathological (i.e., total AET > 6%) or borderline day (i.e., total AET 4–6%), thus falling into a gray area (i.e., inconclusive or borderline). On the other hand, when using the DMS thresholds and definitions [12], 45 (39.5%) patients had a normal DMS on both days, and 69 (60.5%) presented with pathological acid exposure (54 [47.4%] for one day of the study and 15 [13.2%] for both days). Thus, with a 48-h study, the proportion of cases that were classified as normal was significantly lower using the total AET alone than when using the DMS (22.8% vs. 39.5%, *p* < 0.05), and using the total AET underdiagnosed several patients presenting with both days of pathological reflux by DMS (0% vs. 13.2%, *p* < 0.05). Table 2 and Fig. 2 summarize the proportion of patients falling into each diagnostic category according to the employed criteria.

**Table 2:** Proportion of patients falling into each diagnostic category according to the employed diagnostic method

Criteria defining pathological acid exposure	Cohort (*n* = 114)
DeMeester score (≥ 14.73)	
Both days normal [Normal]	45 (39.5)
One day normal and one pathological [Pathological]	54 (47.4)
Both days pathological [Pathological]	15 (13.2)
Acid exposure time (≥ 6%)	
Both days normal [Normal]	26 (22.8)
One day normal and one borderline [Borderline]	38 (33.3)
One day normal and one pathological [Borderline]	26 (22.8)
Both days borderline [Borderline]	14 (12.3)
One day borderline and one pathological [Borderline]	10 (8.8)

Data presented as no. (%). Brackets indicate diagnostic category

*AET* acid exposure time

**Fig. 3:**
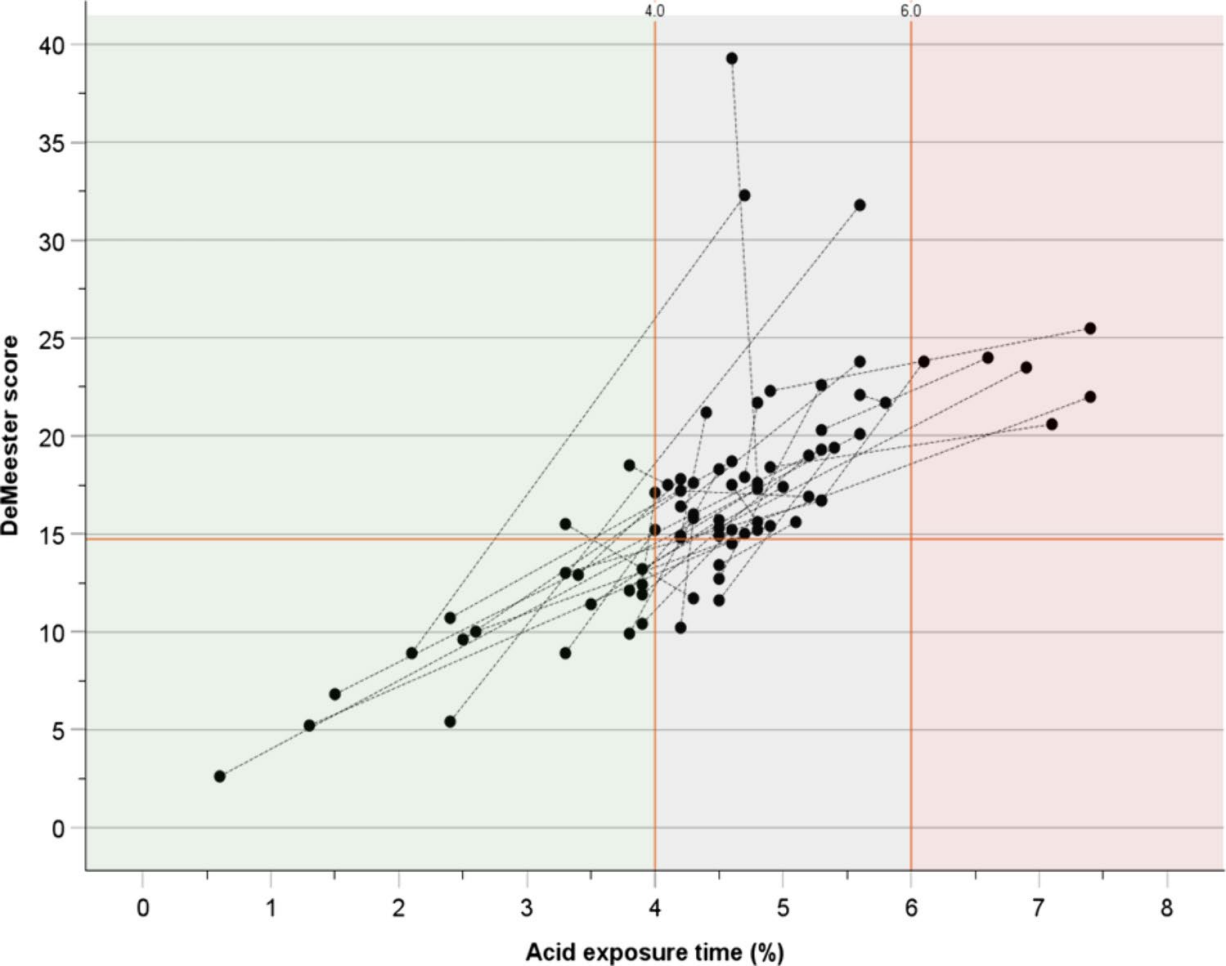
DeMeester score and total acid exposure time (AET) of individual days of analysis (*n* = 78) among the development cohort (*n* = 39). The dotted lines connect the results of both days of study for each subject. Based on total AET criteria, 20 (51.3%) patients had one normal (light green area) and one borderline day (gray area), 6 (15.4%) had one borderline and one pathological day (pink area), and 13 (33.3%) had a borderline AET on both days of study (Color figure online)

To confirm this misclassification within the borderline area, we proceeded to identify incongruent study days (i.e., a single day of analysis with a normal or borderline total AET [< 6%] but pathological DMS [≥ 14.73]). We found a total of 48 (21.1%) days of monitoring with these characteristics retrieved from 39 (34.2%) patients, thus resulting in 78 days for analysis (i.e., the development sample). The baseline characteristics of the patients included in the development sample are summarized in Table 1. Among this subgroup, using total AET thresholds, 20 (51.3%) patients had one normal and one borderline day, 13 (33.3%) had two borderline days, and 6 (15.4%) had one borderline and one pathological day (Fig. 3). Moreover, besides the duration of the longest reflux episode, there was no significant intra-subject variability in terms of pH metrics between day 1 and 2 (Supplementary Material S2), thus confirming that the currently used total AET thresholds (i.e., cutoffs) are more likely than the intra-subject variability of pH metrics to explain the observed misclassification of borderline results.

### Does supine position matter?

To evaluate if supine AET was relevant to the misclassification, we analyzed all the days of registry (*n* = 228) from the cohort and created a cross-tabulation using the DMS and total AET definitions (Table 3). The proportion of individual days of analysis falling into the different diagnostic categories differed significantly according to the method employed (*p* < 0.05); thus, confirming the misclassification. Furthermore, although a strong positive correlation was observed between DMS and total AET (*r*_*s*_ = 0.954 [95% CI 0.941, 0.965], *p* < 0.05), the mean upright AET dramatically increased across the defined AET categories (normal, 3.3 ± 3.2; borderline, 5.7 ± 3.1; and pathological, 9.6 ± 4.7) but the mean supine AET did not (normal, 4.9 ± 1.4; borderline, 4.7 ± 5.6; and pathological, 4.5 ± 4.9). Notably, the variance of the supine AET among days of registry with negative DMS and total AET < 4% (*n* = 114) was lower than the variance presented during upright AET [0.7 ± 1.0 vs. 3.2 ± 2.0; variance ratio, 0.25 (95% CI 0.17, 0.36)]. The summary of upright and supine AET by diagnostic category is presented in Table 3.

**Table 3:** Cross tabulation of individual days of analysis (*n* = 228) according to current diagnostic criteria (i.e., DeMeester score and total acid exposure time)

	Total AET < 4%	Total AET 4–6%	Total > 6%
+ DMS (≥ 14.73)	2 (0.9%) Upright AET: 3.3 ± 3.2 Supine AET: 4.9 ± 1.4	46 (20.2%) Upright AET: 5.7 ± 3.1 Supine AET: 4.7 ± 5.6	36 (15.8%) Upright AET: 9.6 ± 4.7 Supine AET: 4.5 ± 4.9
− DMS (< 14.73)	114 (50%) Upright AET: 3.2 ± 2.0 Supine AET: 0.7 ± 1	30 (13.1%) Upright AET: 6.4 ± 1.5 Supine AET: 0.6 ± 1.1	0 (0%) – –

The mean acid exposure time for each position is presented in each category

Continuous data is presented as mean and standard deviation

*AET* acid exposure time, *DMS* DeMeester score

**Fig. 4:**
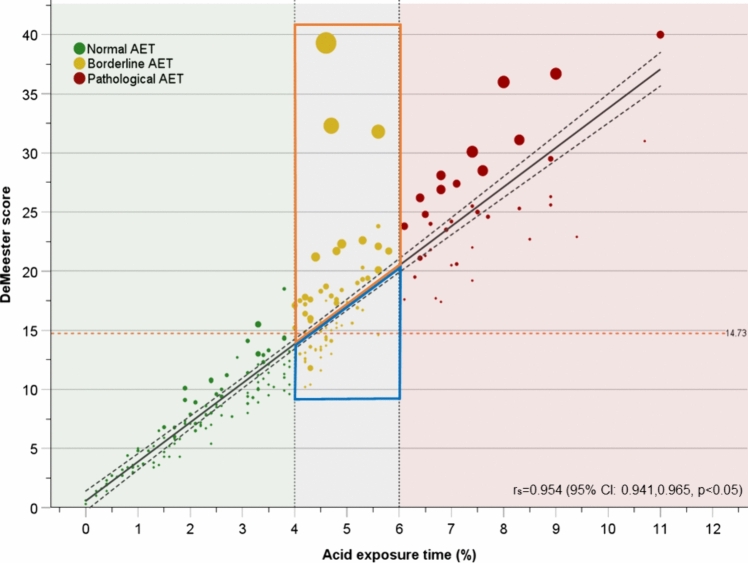
A scatter plot presents the regression for the DeMeester score and total acid exposure time (AET) from the cohort’s individual days of analysis (*n* = 228). The weight of supine AET is represented by the bubble size (i.e., bigger bubble size indicates a higher percentage of supine AET). There are two distinct subsets of borderline studies (i.e., gray area). The first one is characterized by predominant supine reflux (orange polygon) and the second one by predominant upright reflux (blue polygon) (Color figure online)

When incorporating the weight of supine AET into the scatter plot (Fig. 4), 2 distinct subsets were clearly identified within the borderline area (i.e., total AET 4–6%); the first comprised studies presenting predominant supine reflux (i.e., above the regression line; *n* = 38), and the second comprised studies with predominant upright reflux (i.e., below the regression line; *n* = 38). Although the mean total AET was similar between the predominant supine and the predominant upright reflux subsets (4.8 ± 0.5 vs. 4.6 ± 0.4, *p* = 0.109), the mean supine AET (5.7 ± 5.7 vs. 0.5 ± 1.0, *p* < 0.05) and the mean upright AET (5.1 ± 3.1 vs. 6.8 ± 1.6, *p* < 0.05) clearly differed between the subsets. Notably, the difference was more remarkable in terms of supine AET than upright AET. Together, these findings highlight the ability of supine AET to better discriminate between normal and pathological categories than the upright AET and demonstrate that the use of AET alone diminishes the weight that supine AET should receive to improve diagnostic performance.

### Development and internal validation of a new composite score

Because the incongruent study days allow for a better estimation of the weight that supine AET should receive to improve the classification of studies falling into the borderline area, we used the subgroup of 39 patients (*n* = 78 study days) previously described as a development sample. The use of this sample aimed to reduce the potential under- or overestimation of effects caused by a larger sample or the inclusion of several normal or pathological studies. A marginal model indicated that the proportion of variance in the DMS explained by the supine AET in this sample was 56.5%, thus confirming that supine AET has a greater scoring weight in the DMS. Further, the preselected predictors were introduced to create an additive linear model. The supine AET (*β* = 1.286, *p* < 0.05) was a stronger positive predictor than the upright AET (*β* = 0.991, *p* < 0.05). Overall, the model presented an adequate goodness-of-fit and quality (i.e., adjusted *R*^2^ = 0.835, *AIC* = 140.03), and no collinearity was observed between the predictors (*VIF* = 1.27, minimum tolerance: 0.787). The new proposed score (i.e., the pHoenix score) resulted from the simplified equation incorporating the regression coefficients of the predictors and excluding the intercept; hence, the calculation method per day of analysis: pHoenix score=(% upright AET × 0.991)+(% supine AET × 1.286). Thereafter, the model was internally validated via bootstrapping (*B* = 1000). Table 4 incorporates the results from the marginal model as well as the original and internally validated models.

**Table 4:** Marginal, original, and internally validated linear regression models created for the development of a new composite score using both supine and upright acid exposure times

	Original model			Model after internal validation		
Variable	Regression coefficient (95%CI)	Partial correlations	*p*-value	Regression coefficient (95%CI)	Bias	*p*-value
Model 1—marginal model explaining the proportion of variance in DMS caused by supine AET% within the development sample						
Intercept	13.382 (12.298, 14.465)	–	** < 0.001**	–	–	–
Supine AET %	0.948 (0.760, 1.136)	0.756	** < 0.001**	–	–	–
Model 2—model including the preselected predictors in a scoring system based on the explanatory weight of upright and supine AET within the development sample						
Intercept	6.473 (5.076, 7.871)	–	** < 0.001**	6.473 (3.718, 8.457)	− 0.273	** < 0.001**
Upright AET %	0.991 (0.815, 1.167)	0.791	** < 0.001**	0.991 (0.728, 1.370)	0.030	** < 0.001**
Supine AET %	1.286 (1.156, 1.416)	0.915	** < 0.001**	1.286 (1.132, 1.633)	0.045	** < 0.001**

Model 1: DMS = 13.382 + (supine AET% × 0.948). R^2^ = 0.571, Adjusted R^2^ = 0.565, AIC = 214.76

Model 2: DMS = 6.473 + (supine AET% × 1.286) + (upright × 0.991). R^2^ = 0.840, Adjusted R^2^ = 0.835, AIC = 140.03

*95% CI* 95% confidence intervals, *AET* acid exposure time, *DMS* DeMeester score

Bold values indicates statistical significance (*p* < 0.05)

### Thresholds and performance of the pHoenix score

**Fig. 5:**
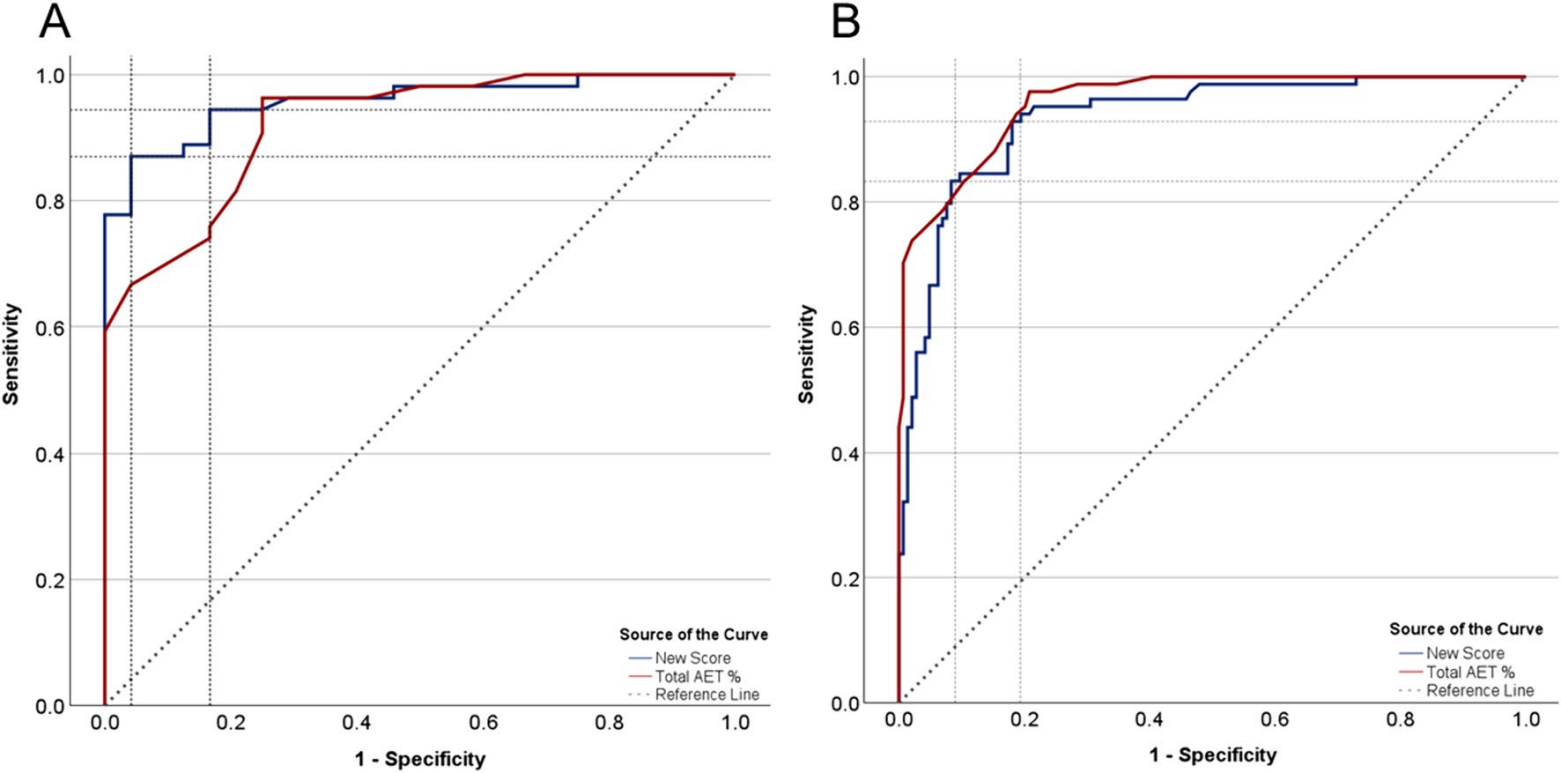
Performance analysis of the pHoenix score using the area under the receiver operating characteristic curve (AU-ROC) and comparison with the total acid exposure time (AET). **A** Assessment of the development sample. **B** Assessment of the entire study cohort

The pHoenix score performance measures and comparison with the use of total AET are presented for both the development sample and the entire cohort in Table 5. The AU-ROC of the pHoenix score in the development sample was 0.957 (95% CI 0.917, 0.998) (Fig. 5A). Further, using the Youden index, an optimal lower cutoff of 7.06 and an optimal upper cutoff of 8.45 were identified and selected. At the 7.06 threshold, the pHoenix score presented an SE of 94.4% (CI 95% 84.6–98.8%) and an SP of 79.2% (CI 95% 57.6–92.9%), whereas at the 8.45 cutoff, the SE was 87% (CI 95% 75.1–94.6%) and the SP was 95.8% (CI 95% 78.9–99.9%). When assessing the performance among the entire cohort, the AU-ROC was 0.934 (95% CI 0.902, 0.966), indicating good discriminatory ability (Fig. 5B), and the SE and SP at both thresholds were preserved (Table 5). Notably, the SE of total AET at the upper threshold (i.e., > 6%) was very low in both the development sample and the entire cohort compared to that of the pHoenix score.

**Table 5:** The predictive performance of the pHoenix score compared to total acid exposure time at each diagnostic threshold

Scoring system	AUC (95% CI)*	Max K–S	*p*-value	Thresholds	SE (95% CI)	SP (95% CI)	NPV (95% CI)	PPV (95% CI)
Total AET %	0.922 (0.863, 0.981)	0.713	** < 0.001**	> 4% > 6%	96.3% (87.3, 99.6) 11.1% (4.2, 22.6)	75.0% (53.3, 90.2) 100% (85.8, 100)	90.0% (69.4, 97.3) 33.3% (31.3, 35.5)	89.7% (81.2, 94.6) 100% (54.1, 100)
pHoenix score	0.957 (0.917, 0.998)	0.829	** < 0.001**	> 7.06 > 8.45	94.4% (84.61, 98.8) 87.0% (75.1, 94.6)	79.2% (57.6, 92.9) 95.8% (78.9, 99.9)	86.4% (67.4, 95.1) 76.7% (62.1, 86.3)	91.1% (82.3, 95.7) 97.9% (87.3, 99.7)
Total AET %	0.960 (0.939, 0.981)	0.768	** < 0.001**	> 4% > 6%	97.6% (91.7, 99.7) 42.9% (32.1, 54.1)	79.9% (71.6, 85.5) 100% (97.5, 100)	98.3% (93.5, 99.6) 75% (71.4, 78.3)	73.2% (66.5, 79.0) 100% (90.3, 100)
pHoenix score	0.934 (0.902, 0.966)	0.750	** < 0.001**	> 7.06 > 8.45	92.9% (85.1, 97.3) 83.3% (77.1, 91.0)	81.3% (73.9, 87.3) 91.7% (85.9, 95.6)	95.1% (89.9, 97.7) 90.4% (85.4, 93.9)	74.3% (67.2, 80.3) 85.4% (77.1, 91.0)

The upper half of the table displays the metrics within the development sample and the lower half displays the metrics for the entire cohort

*AET* acid exposure time, *K–S* Kolmogorov–Smirnov, *NPV* negative predictive value, *SE* sensitivity, *SP* specificity, *PPV* positive predictive value

*A DMS ≥ 14.73 was used as gold standard to define pathological acid exposure

Bold values indicates statistical significance (*p* < 0.05)

### Re-defining diagnostic categories based on the pHoenix score

**Fig. 6:**
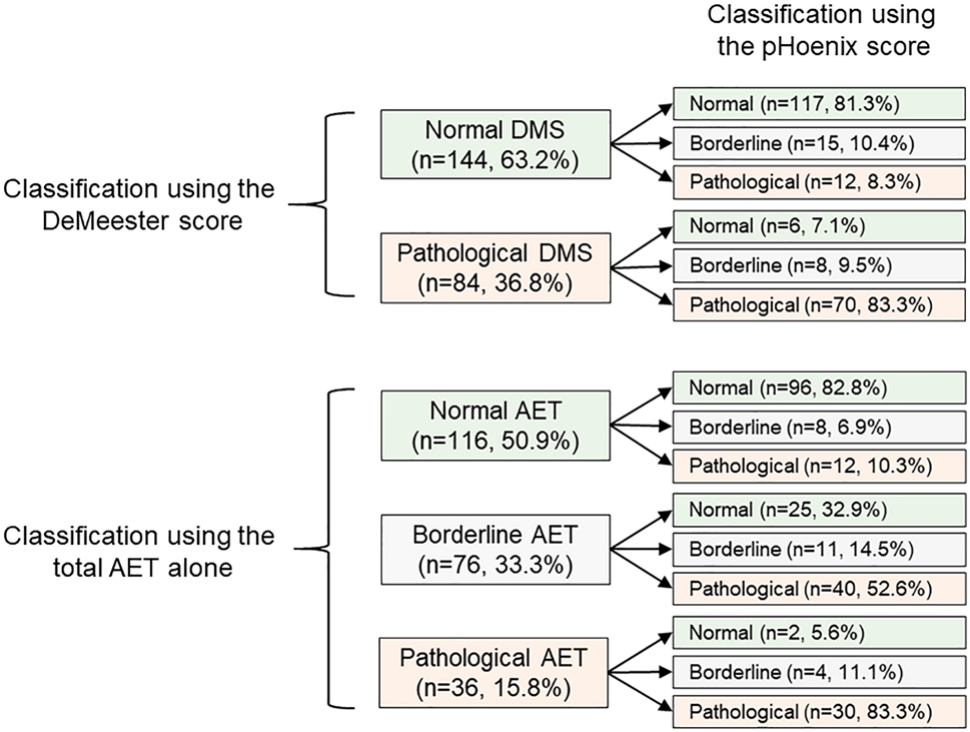
Reclassification of individual days of analysis (*n* = 228) using the pHoenix scoring system. *DMS* DeMeester score, *AET* acid exposure time (Color figure online)

The reclassification of individual days of study (*n* = 228) when using the pHoenix score is presented in Fig. 6 and supplementary Material S3. Notably, the proportion of days initially categorized as borderline by total AET reduced significantly when days were categorized by the pHoenix score (76 [33.3%] vs. 23 [10.1%], *p* < 0.05). Furthermore, we propose a diagnostic classification system using the pHoenix score as follows: (i) normal acid exposure: normal pHoenix score (i.e., < 7.06) on both days of registry, (ii) borderline acid exposure: normal pHoenix score on one day and borderline score (i.e., 7.06–8.45) on one day or two days with a borderline score, and (iii) pathological acid exposure: at least one day with a pathological pHoenix score (i.e., > 8.45).

**Fig. 7:**
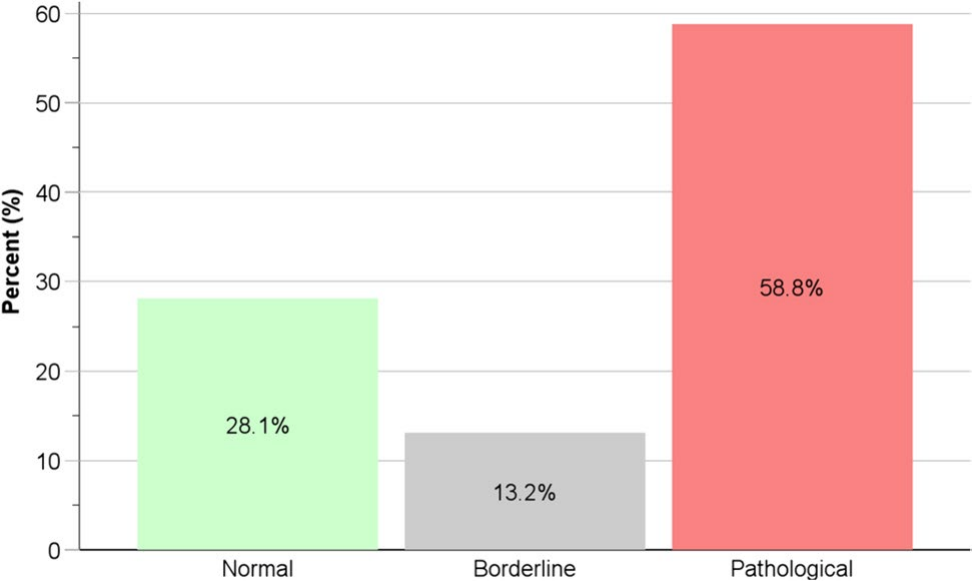
Classification of patients using the pHoenix scoring system (*n* = 114). A complete tabulation is presented in Supplementary Material S4

When using the new diagnostic system, 32 (28.1%) patients were classified with normal acid exposure and 67 (58.8%) with pathological acid exposure; only 15 (13.2%) patients had an inconclusive (i.e., borderline) result. This approach reduced the proportion of patients being classified as borderline based on AET alone (13.2% vs. 77.2%, *p* < 0.001), but maintained the proportion of patients classified as normal (28.1% vs. 39.5%, *p* = 0.069) or pathological (58.8% vs. 60.5%, *p* = 0.892) according to the DMS. Figure 7 and Supplementary Material S4 summarize the diagnostic classification using the pHoenix score. Moreover, the baseline and endoscopic characteristics as well as the overall 48-h pH metrics were compared across the diagnostic categories when using total AET alone or the pHoenix score (Table 6).

**Table 6:** Baseline, endoscopic, and 48-h pH metrics compared across the diagnostic categories according to each diagnostic system

	pHoenix score					Total AET		
		Normal (*n* = 32)	Inconclusive (*n* = 15)	Pathological (*n* = 67)	*p*-value	Normal (*n* = 26)	Inconclusive (*n* = 88)	*p*-value
Demographic characteristics								
Age, years, median (IQR)		60 (49–68)	54 (42–70)	53.5 (41–65)	0.298	60.5 (49–68)	54 (42–66)	0.141
Sex, female (%)		24 (75.0)	10 (66.7)	46 (68.7)	0.772	19 (73.1)	61 (69.3)	0.713
BMI, kg/m^2^, median (IQR)		25 (21–28)	27 (23–29)	30 (26–35)^§^*	**0.007**	25 (23–29)	29.1 (25–33)	0.104
Endoscopic and manometric findings, no (%)								
Endoscopic evidence of GERD		0 (0)	1 (6.7)	9 (13.4)^**§**^	0.157	1 (3.8)	9 (10.2)	0.312
Esophagitis LA ≥ B		0 (0)	0 (0)	3 (4.5)	0.696	0 (0)	3 (3.4)	0.340
Barrett’s esophagus		0 (0)	1 (6.7)	6 (9)	0.333	1 (3.8)	6 (6.8)	0.579
Peptic stricture		0 (0)	0 (0)	0 (0)	> 0.999	0 (0)	0 (0)	> 0.999
Hiatal hernia		15 (46.9)*	2 (13.3)	27 (40.3)*	0.155	12 (46.2)	32 (36.4)	0.368
Hiatal hernia > 2 cm		12 (37.5)	2 (13.3)	15 (22.4)	0.079	9 (34.6)	20 (22.7)	0.221
Manometric IEM or AC^^^		3 (27.3)	4 (40)	13 (29.5)	0.781	4 (36.4)	16 (29.6)	0.742
Average 48-h pH monitoring parameters, mean (SD)								
DeMeester score		9.1 ± 1.9	11.5 ± 2.2	17.3 ± 3.9	** < 0.001**	8.6 ± 1.3	15.9 ± 4.4	** < 0.001**
% Total AET		2.7 ± 0.6	3.2 ± 0.7	4.6 ± 0.9	** < 0.001**	2.4 ± 0.3	4.3 ± 1.0	** < 0.001**
% Supine AET		0.5 ± 0.9	0.9 ± 1.2	3.3 ± 2.9	** < 0.001**	0.7 ± 1.0	2.5 ± 2.8	** < 0.001**
% Upright AET		3.7 ± 1.2	4.8 ± 1.4	5.9 ± 2.7	** < 0.001**	3.5 ± 1.0	5.6 ± 2.5	** < 0.001**

*AC* absent contractility, *BMI* body mass index, *GERD* gastroesophageal reflux disease, *IEM* ineffective esophageal motility, *LA* Los Angeles grade of erosive esophagitis, *SD* standard deviation

Bold indicates statistical significance (*p* < 0.05)

*p-value < 0.05 compared to borderline group

^**§**^p-value < 0.05 compared to normal group

^^^Manometric data was available for 65 patients

Bold values indicates statistical significance (*p* < 0.05)

## Discussion

The increasing incidence of GERD in the Western world as well as the understanding of pathophysiological mechanisms (i.e., mechanical defects of the lower esophageal sphincter) [1–4] has led to a general disenchantment with “treating” GERD with acid suppression. This has increased the push for definitive surgical or endoscopic treatment of GERD; however, before treatment, a definitive diagnosis of GERD needs to be objectively established [13, 19, 20]. The introduction of the Bravo pH capsule and the release of Lyon Consensus criteria brought about shifts in diagnostic approaches, particularly the interpretation of studies with ≥ 48 h of monitoring as well as the transition from the composite DMS to the total AET as a standalone diagnostic metric. Notably, our study demonstrated that the total AET alone does not provide sensitive thresholds, and a large proportion of patients with an inconclusive GERD diagnosis (i.e., up to 82.9%) are susceptible to being reclassified using the DMS. This finding points to the need for reevaluation of the current diagnostic criteria and thresholds to optimize pH monitoring in patients. Thus, we developed and internally validated a diagnostic classification system that uses a composite score, the pHoenix score, to address the many concerns of using the total AET alone or the DMS.

The use of total AET alone to diagnose pathological acid exposure has several pitfalls. Ignoring the weight of supine AET as an indicator of the severity of the disease leads to an underdiagnosis of patients with pathological reflux. Supine AET (as opposed to upright AET) has been widely associated with different esophageal manifestations of GERD, such as erosive esophagitis and Barrett’s esophagus [17, 21–23]. The importance of supine AET was also confirmed in our study by the identification of two subsets of pH studies within the borderline area (i.e., predominant upright and predominant supine reflux) that the total AET did not discriminate.

Furthermore, the current diagnostic thresholds of the total AET (i.e., normal, < 4% and pathological, > 6%) also raise concern. As expected, our analysis revealed an overall adequate performance of the total AET when using the DMS as the gold standard (i.e., because the total AET is one of the DMS parameters); however, if carefully reviewed, it is clear that the upper threshold of total AET (i.e., > 6%) lacks adequate sensitivity despite excellent specificity. In other words, the current definitions adequately identify true positive cases but increase the number of false negative cases that fall into the borderline area. On the other hand, the lower threshold (i.e., < 4%) is closer to an optimal cutoff (SE, 97.6%; SP, 79.9%); however, its use alone may increase false positive cases compared to the DMS. This concern has been previously identified. Padua et al. [24] proposed a decrease in the threshold for GERD diagnosis to a total AET cutoff of 2.3% based on the optimal prediction point (SE, 97%; SP, 91%) established within a cohort of 300 patients who underwent 24-h, catheter-based pH monitoring. The authors recognized the lack of endoscopic information as well as the small proportion of patients with borderline results (7.3%); nevertheless, most of these patients were susceptible to reclassification to pathological acid exposure based on the DMS [24].

On the other hand, some aspects should also be considered with using the DMS despite its robustness and wide use. The relative complexity of calculating the DMS and the need for an adequate record of meals and supine periods make this score highly dependent on both patient adherence to recommendations and the need to document meal times accurately. Although efforts to control both factors are made in clinical practice (i.e., adequate patient counseling), this has become cumbersome. It could be argued that the DMS may not be accurate in some instances when using wireless pH studies (≥ 48 h) due to poor patient adherence in reporting meal periods.

Most importantly, the diagnostic classification using the DMS does not entirely recognize the natural history and progression of the disease or the various reflux phenotypes. This dichotomization necessarily results in an uncertain proportion of false negative and false positive cases. To address this concern while recognizing the continuum of the disease and the weight of supine AET, Bell [25] established a borderline range for the DMS (i.e., between 14.7 and 23.4) [25]; however, these cutoffs were based on the current total AET thresholds (i.e., 4 and 6%), and the weight of supine AET within the borderline cases was considered only for studies with supine AET > 8%.

The diagnosis of pathological acid exposure must consider supine and upright AET. Other pH parameters such as the number of long episodes and the duration of the longest reflux episode alone may present some variability according to demographic and clinical factors [9]. Moreover, emerging metrics such as the symptom index (SI) or symptom association probability (SAP) lack adequate association with the severity of acid exposure [26, 27]. For example, in 2022, Frazzoni et al. [26] reported that a large proportion of patients (39%) with normal total AET presented with a positive SAP or SI, which was similar to patients with borderline (38%) or abnormal total AET (49%). Similarly, in 2023, Visaggi et al. [27] found that SI or SAP metrics were similar among patients presenting with evidence of erosive esophagitis Los Angeles grade A, B, or C. Hence, the use of these metrics to discriminate normal from pathological cases or as adjunctive evidence against GERD may not be reliable.

Because the development of the pHoenix score was based on parameters included in the DMS, it may appear to be a modification of the composite DMS rather than a novel metric. However, the use and interpretation of the pHoenix score differs and has additional advantages. Its main strength is the recognition of the spectrum of acid reflux exposure by including a narrow borderline area that weighs supine AET as appropriate. Moreover, its calculation is more straightforward and intuitive than the DMS. Nevertheless, some concerns around the use of the pHoenix score may arise. For example, adequate patient compliance to reporting positional changes is critical; however, this can be easily addressed with adequate patient counseling, or alternatively, by incorporating a gyroscope to the pH capsule.

On the other hand, the proposed diagnostic system for 48-h studies using the pHoenix score establishes a pathological diagnosis for all individuals with at least one day of pathological acid exposure (i.e., > 8.45). This was based on the study by Ayazi et al. [12] that found a positive DMS score on either the first or second day was the most accurate method to define pathological reflux in adequately selected patients (i.e., with clinical suspicion of GERD). This is justified because most of the normal and pathological cases remain in the same diagnostic category after repeat testing (i.e., even 10 days apart) [28] and on different days within the same study [12, 14]. We believe that the adoption of the pHoenix score will optimize diagnostic resources and sort out the troublesome criteria proposed by the Lyon Consensus [13].

Our study has some limitations including a single-center, retrospective design; however, most of the data were collected prospectively, and a trained interpreter reviewed all of the pH-monitoring studies to guarantee the adequacy of the data. Other potential limitations include the relatively small size of the development sample and a moderately sized cohort for the performance assessment; however, we reached a maximum sample size by convenience. Furthermore, it is unknown how many patients were scheduled to undergo pH testing but did not get a Bravo capsule placed due to a long segment of Barrett’s esophagus or significant esophagitis, which may have decreased the number of patients with endoscopic findings of GERD but borderline total AET. In addition, the development of the pHoenix score as well as the performance analyses did not consider “adjunctive evidence of GERD” or other pH metrics due to the limited evidence supporting adequate discriminatory ability of other tools. Finally, although we internally validated the score, all of the data for the development and validation are from the same center, thus limiting its generalizability. External validations and studies, including Western and Eastern populations, are highly desirable to determine the utility of the proposed score to guide patient management and predict clinical outcomes.

## Conclusion

In summary, our study underscores the limitations of relying on total AET for GERD diagnosis. Current definitions of GERD based on this metric alone disregard positional variations in acid exposure, and the proposed thresholds by the Lyon Consensus lack adequate sensitivity, which ultimately causes considerable misclassification of patients. Our proposed diagnostic classification system based on the pHoenix score provides a practical framework for GERD diagnosis following 48-h wireless pH-monitoring studies. This system offers a more refined approach and addresses the main concerns of using the total AET alone (i.e., a large number of inconclusive cases caused by ignoring the weight of supine AET) or the DMS (i.e., not recognizing the spectrum of the disease, requiring relatively complex calculations, and absolute patient compliance). Future studies to externally validate the pHoenix score and assess its performance with 24-h pH monitoring are highly desirable.

## Supplementary Information

Below is the link to the electronic supplementary material.

Supplementary file1 (DOCX 174 KB)

## Data Availability

The data analyzed in this study can be queried for future studies; however, it cannot be shared outside of those authorized as research staff per protocol. Access to this dataset requires IRB approval; if needed, direct to the corresponding author.
