# Supplementary material for: pHoenix score: development and validation of a novel approach to decrease the number of inconclusive GERD diagnoses
Source: Surg Endosc. 2024 Aug 27;38(11):6880–93. doi: 10.1007/s00464-024-11105-1 (PMC11525326; doi:10.1007/s00464-024-11105-1)
Supplement: Supplementary file 1 — Supplementary file1 (DOCX 174 KB) [file 464_2024_11105_MOESM1_ESM.docx]

**Supplementary material S1 -** The Transparent Reporting of a multivariable prediction model for Individual Prognosis or Diagnosis (TRIPOD) checklist.

| **Section/Topic** | **Item** |  | **Checklist Item** | **Page** |
| --- | --- | --- | --- | --- |
| **Title and abstract** | | | | |
| Title | 1 | D;V | Identify the study as developing and/or validating a multivariable prediction model, the target population, and the outcome to be predicted. | 1 |
| Abstract | 2 | D;V | Provide a summary of objectives, study design, setting, participants, sample size, predictors, outcome, statistical analysis, results, and conclusions. | 2 |
| **Introduction** | | | | |
| Background and objectives | 3a | D;V | Explain the medical context (including whether diagnostic or prognostic) and rationale for developing or validating the multivariable prediction model, including references to existing models. | 3-4 |
|  | 3b | D;V | Specify the objectives, including whether the study describes the development or validation of the model or both. | 4 |
| **Methods** | | | | |
| Source of data | 4a | D;V | Describe the study design or source of data (e.g., randomized trial, cohort, or registry data), separately for the development and validation data sets, if applicable. | 4-5 |
|  | 4b | D;V | Specify the key study dates, including start of accrual; end of accrual; and, if applicable, end of follow-up. | 4-5 |
| Participants | 5a | D;V | Specify key elements of the study setting (e.g., primary care, secondary care, general population) including number and location of centres. | 5 |
|  | 5b | D;V | Describe eligibility criteria for participants. | 5 |
|  | 5c | D;V | Give details of treatments received, if relevant. | N/A |
| Outcome | 6a | D;V | Clearly define the outcome that is predicted by the prediction model, including how and when assessed. | 6 |
|  | 6b | D;V | Report any actions to blind assessment of the outcome to be predicted. | 7-8 |
| Predictors | 7a | D;V | Clearly define all predictors used in developing or validating the multivariable prediction model, including how and when they were measured. | 5-8 |
|  | 7b | D;V | Report any actions to blind assessment of predictors for the outcome and other predictors. | 7-8 |
| Sample size | 8 | D;V | Explain how the study size was arrived at. | 6 |
| Missing data | 9 | D;V | Describe how missing data were handled (e.g., complete-case analysis, single imputation, multiple imputation) with details of any imputation method. | 6-7 |
| Statistical analysis methods | 10a | D | Describe how predictors were handled in the analyses. | 7-8 |
|  | 10b | D | Specify type of model, all model-building procedures (including any predictor selection), and method for internal validation. | 7-8 |
|  | 10c | V | For validation, describe how the predictions were calculated. | 7-8 |
|  | 10d | D;V | Specify all measures used to assess model performance and, if relevant, to compare multiple models. | 7-8 |
|  | 10e | V | Describe any model updating (e.g., recalibration) arising from the validation, if done. | N/A |
| Risk groups | 11 | D;V | Provide details on how risk groups were created, if done. | N/A |
| Development vs. validation | 12 | V | For validation, identify any differences from the development data in setting, eligibility criteria, outcome, and predictors. | N/A |
| **Results** | | | | |
| Participants | 13a | D;V | Describe the flow of participants through the study, including the number of participants with and without the outcome and, if applicable, a summary of the follow-up time. A diagram may be helpful. | Fig 1 |
|  | 13b | D;V | Describe the characteristics of the participants (basic demographics, clinical features, available predictors), including the number of participants with missing data for predictors and outcome. | 8 – Tab1 |
|  | 13c | V | For validation, show a comparison with the development data of the distribution of important variables (demographics, predictors and outcome). | 8 – Tab1 |
| Model development | 14a | D | Specify the number of participants and outcome events in each analysis. | 8-12 |
|  | 14b | D | If done, report the unadjusted association between each candidate predictor and outcome. | 11 – Tab4 |
| Model specification | 15a | D | Present the full prediction model to allow predictions for individuals (i.e., all regression coefficients, and model intercept or baseline survival at a given time point). | Tab 11 |
|  | 15b | D | Explain how to the use the prediction model. | 12 |
| Model performance | 16 | D;V | Report performance measures (with CIs) for the prediction model. | 11 – Tab5 |
| Model-updating | 17 | V | If done, report the results from any model updating (i.e., model specification, model performance). | N/A |
| **Discussion** | | | | |
| Limitations | 18 | D;V | Discuss any limitations of the study (such as nonrepresentative sample, few events per predictor, missing data). | 15 |
| Interpretation | 19a | V | For validation, discuss the results with reference to performance in the development data, and any other validation data. | 15 |
|  | 19b | D;V | Give an overall interpretation of the results, considering objectives, limitations, results from similar studies, and other relevant evidence. | 12-15 |
| Implications | 20 | D;V | Discuss the potential clinical use of the model and implications for future research. | 12-15 |
| **Other information** | | | | |
| Supplementary information | 21 | D;V | Provide information about the availability of supplementary resources, such as study protocol, Web calculator, and data sets. | N/A |
| Funding | 22 | D;V | Give the source of funding and the role of the funders for the present study. | 2 |

*Items relevant only to the development of a prediction model are denoted by D, items relating solely to a validation of a prediction model are denoted by V, and items relating to both are denoted D;V. We recommend using the TRIPOD Checklist in conjunction with the TRIPOD Explanation and Elaboration document.

**Supplementary material S2 -** Summary of pH monitoring metrics of the development sample (i.e., patients with abnormal DeMeester score but normal or borderline total acid exposure time, n=39).

| **Parameters** | **Day 1** | **Day 2** | **p-value** |
| --- | --- | --- | --- |
| DeMeester score | 17.07 ± 5.1 | 16.03 ± 6.7 | 0.487 |
| Total AET (%) | 4.5 ± 1.3 | 4.4 ± 1.2 | 0.787 |
| AET during upright position (%) | 5.4 ± 3.5 | 6.3 ± 3.5 | 0.257 |
| AET during supine position (%) | 3.7 ± 3.5 | 3.0 ± 5.7 | 0.580 |
| Number of reflux episodes | 26 ± 13.1 | 27.4 ± 16.8 | 0.544 |
| Duration of longest reflux episode, min. | 19.6 ± 12 | 12.9 ± 9.6 | **0.011** |
| Continuous data is presented as mean and standard deviation. **Abbreviations: AET:** acid exposure time; **min:** minutes. | | | |

**Supplementary material S3 –** A) Scatter plot presenting current classification for individual days of study (n=228) using the DeMeester score ([DMS], y-axis) and the total acid exposure time ([AET], x-axis), the bubble size represents the percentage of supine AET in each day of the registry and the colors the classification using total AET. B) Modeling of the pHoenix score presenting the classification of individual days of study (n=228) using the pHoenix score compared to DMS (y-axis) and total AET (x-axis); the color zones represent the areas where most of the studies for each category fall into (green: normal, grey: borderline and red: pathological). C) Reclassification for individual days of study (n=228) using the pHoenix score compared to the current classification based on total AET. Of note, there is a reduction in the borderline area (grey zone); hence, a reduction of days of analysis that would be considered inconclusive; similarly, studies presenting a relatively greater supine AET are more prone to be classified as pathological when using the pHoenix score.


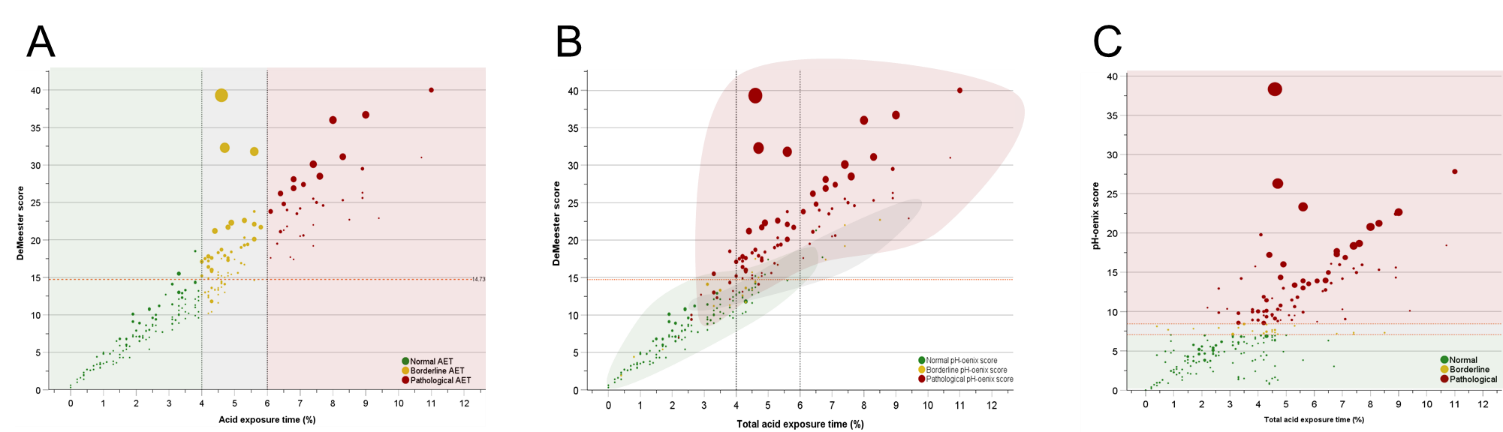


**Supplementary material S4 –** Cohort (n=114) diagnostic classification using the proposed system based on the pHoenix score.

| **Patient classification based on the pHoenix score** | **Cohort**  **(n=114)** |
| --- | --- |
| Both days normal [Normal], (%) | 32 (28.1) |
| One day normal and one borderline [Borderline], (%) | 14 (12.3) |
| Both days borderline [Borderline], (%) | 1 (0.9) |
| One day normal and one pathological [Pathological], (%) | 46 (40.4) |
| One day borderline and one pathological [Pathological], (%) | 7 (6.1) |
| Both days pathological [Pathological], (%) | 14 (12.3) |
| Summary by categories:   - Normal acid exposure: 32 (28.1%) - Borderline/Inconclusive diagnosis: 15 (13.2%) - Pathological acid exposure: 67 (58.8%) | |
